# Therapeutic Physical Exercise Post-Treatment in Breast Cancer: A Systematic Review of Clinical Practice Guidelines

**DOI:** 10.3390/jcm9041239

**Published:** 2020-04-24

**Authors:** Alicia del-Rosal-Jurado, Rita Romero-Galisteo, Manuel Trinidad-Fernández, Manuel González-Sánchez, Antonio Cuesta-Vargas, Maria Ruiz-Muñoz

**Affiliations:** 1 Department of Physiotherapy, Institute of Biomedicine of Málaga (IBIMA), Clinimetric Group (F-14), Chair of Physiotherapy and Disability, Faculty of Health Sciences, Andalucía Tech, University of Málaga, 29071 Málaga, Spain; alidelrosal@gmail.com (A.d.-R.-J.); m.trinidad@uma.es (M.T.-F.); acuesta@uma.es (A.C.-V.); 2 Department of Physiotherapy, Faculty of Health Sciences, Andalucía Tech, University of Málaga, 29071 Málaga, Spain; 3 School of Clinical Sciences of the Faculty of Health, Queensland University of Technology, Brisbane, QLD 4059, Australia; 4 Department of Nursing, Institute of Biomedicine of Málaga (IBIMA), Clinimetric Group (F-14), Chair of Physiotherapy and Disability, Faculty of Health Sciences, Andalucía Tech, University of Málaga, 29071 Málaga, Spain; marumu@uma.es

**Keywords:** breast cancer, physical exercise, systematic review, clinical practice guideline

## Abstract

Advances achieved in diagnosis and improvements in treatment for breast cancer have resulted in a favourable survival rate. Therapeutic physical exercise (TPE) is presented as an intervention strategy that seeks to improve the functional capabilities of the subject. To analyse if clinical practice guidelines recommend therapeutic physical exercise to reduce the adverse effects of treatment in breast cancer survivors, and on what level of scientific evidence are these recommendations based. This systematic review was prepared by searching nine electronic databases to identify eligible studies. Thirteen met the criteria for inclusion. The Appraisal of Guidelines for Research and Evaluation (AGREE II) scale was used to analyse the quality of Clinical Practice Guideline (CPGs). The percentages obtained ranged between 30.07% and 75.70%. Specifically, the highest degree of evidence could be found in the application of TPE to offset adverse effects leading to effects such as: an increase in the quality of life, fatigue reduction, and reduction in body weight alterations. TPE is presented as an optimal intervention strategy to alleviate the negative effects that patients with breast cancer suffer as a result of the treatments received. The level of evidence that supports this claim is very strong for the majority of the side effects analysed. However, this evidence is not always included in the clinical practice guidelines.

## 1. Introduction

Breast cancer is a pathology whose highest annual incidence rate is found in industrialised countries [1]. Furthermore, advances achieved in diagnosis and improvements in treatment have created a favourable survival rate of 89.2% five years after diagnosis [2].

However, it is a pathology whose adverse effects and treatment processes mean that the quality of life of patients is altered, which leads to a series of health needs that generate certain costs in society. Hospitalisation, drug administrations, and sick leave, which the disease entails, are some examples of these costs [1].

The increase in healthcare costs in patients who are survivors of breast cancer is usually derived, to a large extent, from compensating for the adverse effects resulting from the treatment of breast cancer [3]. The most frequent adverse effects suffered by breast cancer survivors as a consequence of treatment are: decreased quality of life, increased morbidity, increased risk of recurrence, fatigue, pain, stress, depression, anxiety, peripheral neuropathy, reduced range of motion of the shoulder, upper limb numbness, upper limb tingling, musculoskeletal changes, and lymphedema [3,4], among others.

Therapeutic physical exercise (TPE) is presented as an intervention strategy that seeks to improve or maintain the functional capabilities of the subject [5]. Exercise is a useful and necessary tool for the physical and mental well-being of the general population [6]. But it is essential in patients who have a chronic pathology causing reduced quality of life is. Exercise generates an improvement at a systemic level; it produces cardiovascular, muscular-skeletal, and endocrine benefits, among others. In cancer, the adverse effects produced both by the treatments and by the disease itself can be diminished or attenuated by TPE [7,8,9]. This therapeutic strategy can be used for both pre-treatment and post-treatment, showing success in achieving an improvement in quality of life that is reduced in these patients [6].

Clinical practice guidelines (CPGs) are reference documents for clinical professionals that include recommendations on the care, diagnosis, and appropriate treatment of certain diseases [7,10]. Clinical practice guidelines are based on studies of high scientific evidence with the aim of improving clinical practice. Therefore, they are tools that are used to improve the quality of care offered to the patient in order to improve clinical outcomes [7].

One of the objectives of this study was to analyse if clinical practice guidelines recommend therapeutic physical exercise to reduce the adverse effects of treatment in breast cancer survivors, and on what level of scientific evidence are these recommendations based. In addition, it also aimed to specify the adverse effects of breast cancer that can be reduced by intervention with a TPE programme.

## 2. Methods

This systematic review was prepared according to the protocol registered in PROSPERO: International prospective register of systematic reviews, with reference number CRD42018103258, and has been submitted in accordance to the Preferred Reporting Items for Systematic Reviews and Meta-Analyses (PRISMA) statement.

### 2.1. Search Strategy

The following databases were used for the literature search: PubMed, ScienceDirect, Google Scholar, CINAHL, PEDro, Scopus, National Guideline Clearinghouse (NGS), and National Institute for Health and Clinical Excellence (NICE). The following keywords were used for the search: “Breast cancer”, “Physical treatment”, “Exercise”, and “Guideline”. Keywords were combined using the Boolean operators “AND” and “OR”.

### 2.2. Study Selection

Initially, two independent investigators (AdRJ and MTF) analysed and selected studies that could potentially be included based on title and abstract. In the case of disagreement, a third investigator (MGS) issued a judgment. The full texts of the selected articles were evaluated in the same manner. For the selection of clinical practice guidelines the following inclusion criteria were used: clinical practice guidelines for breast cancer, published in Spanish, French, English, Italian, and/or Portuguese, published before March 2020 and, in case of successive editions, the latest version was selected. Exclusion criteria used were guidelines that did not include TPE as part of the post-intervention strategy and reports not on humans.

### 2.3. Assessment of Study Characteristics

#### 2.3.1. Quality

For the evaluation of the various selected documents, the Appraisal of Guidelines for Research and Evaluation (AGREE II) was used to determine the quality of these CPGs. This scale evaluates the quality of the various guidelines and recommendations offered by the CPG, it also provides a methodological strategy for the development of various guidelines and instructs on how the information given by the CPGs should be transmitted [11].

Independently, two authors (AdRJ and MTF) examined the full text of the clinical guidelines included in the study to analyse the risks of bias according to the AGREE II [11]. In order to reach a consensus, if there was a discrepancy in the evaluation of the documents, a third investigator’s (AICV) judgment was used.

#### 2.3.2. Levels of Evidence

Once the CPGs that meet the inclusion/exclusion criteria were identified, with the intention of being able to compare the level of evidence on which the recommendations made by each CPG are based, and in order to unify the different levels of evidence shown in the selected CPGs, a scale of evidence was developed following the literature search [12]. This scale allows comparison between the different results extracted from the CPG (Table 1).

#### 2.3.3. Degrees of Recommendation

To establish the degree of recommendation of the CPGs, a colour code system was established in order to facilitate the capture of the necessary information [12] (Table 2). 

## 3. Results

**Figure 1:**
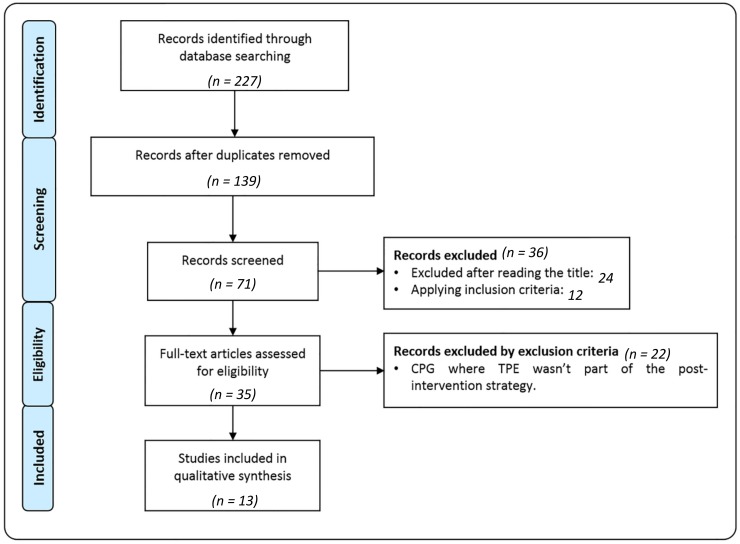
Flowchart of search results and filtering of the documents selected in this study.

Thirteen document were selected. For more details of the search results see Figure 1. The percentages obtained ranged between 30.07% and 75.70% (Table 3). The following table shows the score presented by each of the CPGs in the different domains that the AGREE II scale describes.

To summarize and be able to compare the level of evidence and the degrees of recommendations made by the different CPGs regarding the recommendation of TPE as an intervention to reduce the adverse effects derived from treatment in surviving patients with breast cancer, Table 4 shows in detail the level of evidence and the degrees of recommendation extracted from the different CPGs for each of these adverse effects. Analysing Table 4, it is observed that only for anxiety was there consistency between the grade of recommendation and level of evidence, while the usual trend was disparity both in the levels of recommendation and in the degrees of evidence when comparing the different CPGs. Specifically, the highest degree of evidence could be found in the application of TPE to offset adverse effects such as: an increase in the quality of life, fatigue reduction, and reduction in body weight alterations (Table 4).

## 4. Discussion

Several of the adverse effects caused by treatment received during breast cancer could be reduced by applying TPE as a therapeutic strategy. However, it was also observed that the different CPGs that were analysed in the present study showed disparity regarding the levels of evidence and degrees of recommendation for TPE as a strategy to diminish the adverse effects that the treatment of breast cancer entails (Table 4).

### 4.1. Mortality Reduction

Mortality is one of the most important adverse effects for breast cancer survivors; however, only one CPG proposed TPE as a strategy to achieve reduced mortality from breast cancer. Despite the moderate degree of evidence that this CPG presented [13], the degree of recommendation to use TPE to reduce mortality in breast cancer survivors is very low. Systematic reviews with a high degree of evidence (grade A), demonstrate how the use of a TPE programme based on resistance training reduces several adverse factors in these patients, achieving a reduction in these factors, and decreasing the mortality rate by breast cancer [3].

### 4.2. Recurrence Risk Reduction

The reduction of recurrence of a new breast cancer when performing TPE generates some controversy in the different CPGs. Two of the CPGs contemplated TPE to reduce the likelihood of recurrence of a new breast cancer [17,24], but neither recommended nor disapproved of this strategy. Despite this, we found that the 2012 National Comprehensive Cancer Network (NCCN) CPG [17] presented a high degree of evidence (grade A). This highlights the high degree of recommendation that another CPG presents [13], which generates controversy with the previous two; although its degree of evidence was low (grade C).

However, a document with a high degree of evidence (grade A), demonstrated how TPE decreases certain pro-inflammatory markers and with it the risk of recurrence of a new breast cancer [25]. A systematic review from the year 2017 reinforced this statement by showing that there is less risk of suffering from breast cancer again when performing TPE [26].

### 4.3. Increase in the Quality of Life

Regarding the increase in the quality of life of breast cancer survivors, only two CPGs mentioned the practice of TPE as a tool to achieve this increase. The degrees of evidence presented by these CPGs [13,14] were grades B and A (moderate and strong), respectively. The controversy is evident in the degree of recommendation of the practice, since we find on the one hand that [13] neither recommended nor disapproved of this therapeutic strategy and, nevertheless [14], show that using TPE to increase the quality of life for oncology patients is highly recommended. This disparity in the level of recommendations in the CPGs reveals the outdated nature of the CPG published in 2003 [13], since current high quality evidence documents show how TPE is a useful strategy to achieve an increase in the quality of life of survivors of breast cancer [27]. With the practice of TPE, certain pro-inflammatory markers that are associated with a decrease in the quality of life are decreased; so that with their reduction, an increase in the quality of life of breast cancer patients is achieved [25]. One intervention that has been effectively achieved to achieve slight improvements in quality of life is progressive resistance training [28]. However, maintaining adequate levels of physical activity, favoring the maintenance of high levels of quality of life, both in breast cancer survivors [28,29], and in other patient profiles [30,31,32,33], as well as in a healthy population [34,35] remains necessary.

### 4.4. Pain Reduction

Pain is a common side effect of all breast cancer treatments. Only 3 of the 13 CPGs analysed recommended using a TPE programme to reduce this adverse effect. The grades of recommendation presented by these CPG ranged from moderately recommendable [19,21], to highly recommended [21]. The evidence presented by them ranged from grade B (moderate) to grade A (strong) [19,21]. With a strong degree of evidence (grade A), a systematic review [36] demonstrated the effectiveness of TPE using a specific technique, such as Pilates or yoga, to achieve a reduction of post-treatment pain in breast cancer survivors.

### 4.5. Fatigue Reduction

The physical and mental fatigue that this pathology causes in patients is an adverse effect that generated significant disparity in the different CPGs analysed. There was no clear consensus regarding the level of evidence or the degree of recommendation that they presented for the application of TPE to reduce fatigue in breast cancer survivors. The degrees of recommendation that we found ranged between neither recommending nor disapproving of [14,16] to highly recommending TPE [21], including those indicating it was moderately recommendable [13,15,18,21]. We highlight two CPGs [15,16] for not presenting degrees of evidence in said recommendation. On the other hand, Caroly D. Runowicz [21] presented a degree of evidence that was grade A (strong) in terms of performing TPE to reduce fatigue in breast cancer survivors. With a strong degree of evidence (grade A) a systematic review [25] reinforced this recommendation by demonstrating that TPE presents improvements in terms of reducing fatigue in breast cancer survivors. Similarly, another document with grade A evidence [27], ensured that TPE is a safe strategy to reduce this adverse effect. Only one of the CPGs analysed [18], presented a low degree of evidence (grade c) and could be considered outdated due there is strong evidence that this therapeutic strategy decreases fatigue in breast cancer survivors.

### 4.6. Lymphedema Reduction

The appearance of lymphedema is a frequent adverse effect in the ipsilateral upper limb after a surgical intervention [37]. The recommendations for the use of a TPE programme with the aim of reducing or preventing the appearance of lymphedema, given by the various CPGs analysed, were varied in terms of the degrees of evidence and recommendation. The evidence found in them was grade B (moderate) [13,21] and grade D (very low) [18,21]. Three of the 7 CPGs that recommended this strategy did not show degrees of evidence [15,16,20]. Regarding the levels of recommendation of the different CPGs, we found a great diversity. With a moderate recommendation grade [20,21], several CPGs suggested TPE to reduce lymphedema. Three CPG were found [13,16,18] that neither recommended nor disapproved of TPE to generate benefits in terms of the reduction of lymphedema. We highlight a CPG with a high grade of recommendation published in 2008 [15]. This disparity, both in the degrees of evidence and in those of recommendation, must be resolved; thus, creating a clear and concise consensus on whether TPE is an effective strategy to achieve the reduction of this adverse effect. Scientific evidence, within two systematic reviews with a level of evidence (grade A) [3,28], shows that TPE is an effective and safe tool to reduce lymphedema in breast cancer survivors. These reviews [3,28] affirmed that there is no risk, when performing a TPE programme of resistance, of the appearance or increase of lymphedema. By reducing the presence of this adverse effect, it is possible to increase the strength in the affected upper limb. This increase in strength is associated with an increase in the quality of life of the survivors and a decrease in the probability of post-breast cancer death [3,28].

### 4.7. Reduction of Musculoskeletal Disorders

The recommendations offered by a CPGs analysed in the present study [21] presented similar grades of recommendation, indicating TPE is moderately recommendable, and grade of evidence (grade B). A systematic review published in 2017, with grade A evidence (strong grade), showed that there is evidence that TPE achieves an improvement in musculoskeletal alterations presented by breast cancer survivors. Specifically, resistance training was the most effective to achieve this benefit [21]. Despite the solid evidence for this therapeutic strategy, there is no clear consensus on the type of programme best suited for breast cancer patients. Future studies are necessary to determine the optimal parameters of training intensity and duration [3]. Applying this therapeutic tool can achieve an increase in muscular strength, which is fundamental for the patients. A systematic review with grade A evidence (strong grade) demonstrated the effectiveness of this claim [27].

### 4.8. Increased Range of Motion of the Shoulder

The reduction of the joint range of the shoulder ipsilateral to the surgical intervention, in breast cancer, is another of the adverse effects that can be diminished with the application of a TPE programme. Only 2 of the 13 CPGs analysed recommended applying this strategy with a moderate degree. We emphasise the CPG [20] that presented a high degree of evidence (grade A) for the recommendation to apply TPE to increase the range of movement of the shoulder. The scientific evidence reinforced this affirmation, in a systematic review with a strong degree of evidence (grade A) [37]. This review showed how using TPE, consistently applying a Pilates programme of 45 min per session and three times per week, was useful to increase the range of movement in the shoulder [36].

### 4.9. Reduction of Body Weight Alterations

The changes suffered both from the disease itself and the treatments received on the body weight of breast cancer patients are adverse effects for which it is recommended to perform TPE. These recommendations are very different in the various CPGs analysed. The degrees of recommendation found in them were: neither recommended nor disapproved of [12,13,21] or highly recommended [24]. Note that despite the low degree of recommendation [13,14], these CPGs presented a strong level of evidence (grade A). Systematic reviews published in 2017 [3,25], with a high level of evidence (grade A), demonstrated how TPE reduces body weight alterations in breast cancer survivors. By performing TPE, certain pro-inflammatory markers and body fat are reduced [25]. With resistance training, breast cancer survivors could increase their lean mass and decrease their fat mass, thereby decreasing this adverse effect [3].

### 4.10. Reduction of Gastrointestinal Alterations and Hot Flushes

The gastrointestinal alterations suffered by breast cancer survivors can be reduced by applying a TPE programme. Only two of the analysed CPGs recommended TPE to a moderate degree [13] or highly recommended it [15] to reduce these alterations. These recommendations did not present a degree of evidence, so future studies that determine the scientific evidence that TPE presents regarding the reduction of gastrointestinal alterations in breast cancer survivors would be interesting.

The hot flashes caused by post-treatment of breast cancer is an adverse effect that only one CPG contemplated could be reduced by applying TPE. A CPG [18] presented a low degree of evidence (grade D) and neither recommended nor disapproved of TPE to reduce the hot flashes suffered by these patients.

### 4.11. Reduction of Bone Alterations

There is a clear consensus among the different CPGs analysed on the degree of recommendation for TPE to reduce bone alterations in breast cancer survivors. All of them neither recommended nor disapproved of TPE to reduce this adverse effect. Note that, despite the low grade of recommendation, Carolyn D. Runowicz’s CPG [21] presents a high grade of evidence (grade A).

### 4.12. Reduction of Neuropathies

Only two of the CPG analysed in this study contemplated the application of TPE to reduce the neuropathies caused by the treatments received or by the disease itself. The grades of recommendation vary between moderately recommendable [13] and highly recommended [21]. Only one of the CPGs offered the level of evidence, and it was low grade (grade C).

### 4.13. Reduction of Stress, Anxiety, and Depression

Stress, anxiety, and depression are common side effects presented by breast cancer survivors. Several CPGs contemplated in their recommendations the use of TPE to reduce such adverse effects. There was a similarity in the degrees of recommendation and evidence of the different CPGs about this therapeutic strategy. Some CPGs [13,19,23] had a high grade of recommendation and degree of evidence (grade B) for the use of TPE to reduce these adverse effects. We emphasise one CPG [13] that neither recommended nor disapproved of TPE to reduce the depression that breast cancer survivors may suffer.

### 4.14. Other Adverse Effects

Survivors of breast cancer, as a consequence of the interventions and treatments to which they have been subjected, present numerous adverse effects, many of which were not contemplated by the different CPGs included in the present study to be reducible with a TPE programme. Some of them, such as cardiorespiratory alterations or inflammatory processes, could be reduced by applying a TPE programme. A systematic review with a high grade of evidence (grade A) [27], showed that TPE is a useful strategy to prevent cardiovascular diseases that arise post-breast cancer. On the other hand, this therapeutic strategy reduces certain inflammatory markers and with it numerous adverse effects that the treatment of this pathology causes [25].

### 4.15. Impacts of Types of Training on Different Outcomes

It has been observed how TPE may improve the following outcomes: 1. Recurrence risk reduction; 2. Increase quality of life; 3. Reduction of gastrointestinal disorders; 4. Neuropathy reduction; 5. Stress; 6. Depression; 7. Anxiety; 8. Pain reduction; 9. Fatigue reduction. 10. Increase in range of movement of the shoulder. TPE may not improve or have controversial impacts on the following outcomes: 1. Hot flashes reduction; 2. Reduction of body weight alterations; 3. Reduction of bone alterations; 4. Lymphedema reduction. (Table 4). However, there are several types of training that could be undertaken by breast cancer survivors to achieve a reduction in adverse effects. There are some recommendations that are common to all the CPGs consulted: TPE must be individualized. It is necessary to take into account the personal characteristics of the patient, the type of treatment received, and the adverse effects that they present. In addition, the TPE shoud be supervised by a healthcare professional. Studies show that supervised training produces greater gains in patients than those that do not have supervision by a healthcare professional [3].

However, as with healthy subjects, the predominant type of training in the TPE program could determine the magnitude of the improvement and the adverse effect on which it occurs. Below are the most important aspects when planning a predominantly resistance, strength or flexibility training that should be taken into account when planning a TPE protocol.

#### 4.15.1. Resistance

Regarding resistance training, we highlight the duration of the session, which the recommendations of the scientific literature show to be between 30–60 min a day, with moderate to vigorous intensity [13,14,38]. For the recovery of patients to be complete, there should be 2 min between sets of exercises [3]. This type of training should be done at least 3 days a week and a maximum of 5 days a week [13,14]. Resistance training has enormous benefits in terms of reducing musculoskeletal disorders [3]. In breast cancer patients, this training will increase muscle strength, decrease fat mass, and there is no added risk of lymphedema [3,30].

#### 4.15.2. Strength

The main objective of this type of training is to recover the range of motion lost in the shoulder joint post-surgery. A programme for strengthening the upper limb carried out by a physiotherapist is recommended [39]. During the performance of this type of training, patients should not exceed 5 kg of weight; otherwise there will be a risk of lymphedema [37]. To achieve strength training, it has been observed that developing a progressive resistance training program achieves clinically relevant improvements in both upper and lower limbs [27]. It is very important to introduce the force variable as a goal to be developed in any intervention program in patients surviving breast cancer. Loss of strength in the lower limbs is a powerful predictor of all kinds of causes of death [40]. Therefore, it is necessary to avoid, through the rehabilitation program, a reduction of strength in the lower limbs in surviving patients with breast cancer.

#### 4.15.3. Flexibility

This type of training presents its main benefits at the level of joints and soft tissues [38]. The decrease in alterations at the muscular level is of vital importance for articulation of the shoulder; since surgical intervention for breast cancer reduces this range of motion. The main muscles that must undergo this type of training are the flexors, extensors, adductors, and shoulder rotators [37].

There is no pre-established training programme based on the treatment received or the adverse effects experienced by the patient. Some authors recommend the fusion of training styles to obtain the best benefits for survivors of breast cancer [4]. With the completion of a TPE programme based on flexibility, resistance, and aerobic exercises, a greater decrease in pain and increased flexibility and strength can be achieved. However, reductions of fatigue and fat mass are not achieved with a combined TPE programme [4].

The literature proposes the combination of resistance training and the Pilates method three times a week in 50 min sessions. With this, it seems that a greater reduction in musculoskeletal alterations is achieved [36]. In addition to decreasing most of the alterations in the patients, TPE is a tool that benefits the healthcare system; since it reduces costs by minimising the adverse effects of a chronic disease such as breast cancer [41].

### 4.16. Clinical Practice Guidelines Update

CPGs are the reference documents of clinicians for the use and consultation of therapeutic strategies applied to their patients [10,41]. Science advances much faster than the updates that are made in CPGs; so there are certain recommendations that have been outdated from three different perspectives: level of evidence, degree of recommendation, and adverse effects that can be compensated for using TPE as an intervention strategy. Therefore, it is necessary to update the CPGs when there are documents with a high degree of evidence that show progress for a therapeutic strategy. This occurs in certain recommendations that are given to clinicians through CPGs to reduce the adverse effects of breast cancer treatment, including using TPE.

The present study demonstrates how to reduce the probability of suffering a recurrence of breast cancer, increase the quality of life, and reduce fatigue, lymphedema, body weight, bone alterations, and depression; as the recommendations for all these adverse effects are outdated. Most CPGs show a very low grade recommendation for TPE to decrease the effects mentioned above. But current studies show that there is evidence that TPE reduces such effects. Therefore, it will be necessary that both clinical recommendations and scientific evidence advance in parallel, to ensure therapeutic success.

On the other hand, in a paradigm where the evidence acquires increasing clinical relevance, it should be necessary that the healthcare professionals in charge of carrying out the rehabilitation process in patients who survive breast cancer acquire the necessary skills to search, identify and select studies with the most solid evidence so that they can integrate new approaches immediately to their clinical practice without having to wait for the updates of the different CPGs.

### 4.17. Strengths and Weaknesses and Future Research

Among the strengths of the present study is that it proposes a parallel vision for the scientific literature and clinical practice, thereby achieving the most effective treatments for patients. In the same way, a proposal is also made to update the evidence proposed in the different CPGs through the identification of documents with a high level of evidence (grade A systematic reviews and meta-analyses).

However, the present study also presents some limitations that must be taken into account when interpreting the results presented, such as, for example, the exclusion of documents written in a language other than Spanish, French, English, Italian, or Portuguese. Documents published in other languages were not selected in this study.

Future research directions: considering the results observed in the present study, it would be necessary to carry out studies that would consolidate the evidence, as well as better define the effects of the different interventions whose evidence is not sufficiently consolidated or even that is contradictory.

## 5. Conclusions

Therapeutic physical exercise is presented as an optimal intervention strategy to alleviate the negative effects that patients with breast cancer suffer as a result of the treatments received. The level of evidence that supports this claim is very strong for the vast majority of the side effects analysed. However, this evidence is not always included in the clinical practice guidelines, which have been shown to be outdated in many cases as far as TPE intervention in patients surviving breast cancer is concerned. Therefore, it is necessary to increase the frequency of updates of CPGs to avoid this type of document from diminishing in its clinical utility.

## Figures and Tables

**Table 1:** Agreement for the stratification of the levels of evidence.

Level of Evidence	Type of Study	Solidity
**A**	Meta-analysis	Very strong
	Systematic reviews.	Strong
**B**	Randomized clinical trials.	Moderate
	Cohort studies. Case-control studies.	
**C**	Non-analytical or experimental studies	Low
**D**	Opinion of the committee of experts.	Very Low

**Table 2:** Grade of recommendation according to the Oxford Center for Evidence-based Medicine Levels of Evidence (May 2001).

Color According to Recommendation	Meaning
*	Very recommendable
**	Moderately recommendable
†	Not recommended
‡	Controversy (Neither recommended nor Disapproved)

**Table 3:** Results of the evaluation of the clinical practice guidelines selected for the present study evaluated with the Appraisal of Guidelines for Research and Evaluation (AGREE II) scale.

CPG	Doyle, C [13]	Rooney, M [14]	Manchon, P [15]	Murray, N [16]	Gradishar, WJ [17]	Harris, SR [18]	Greenlee, H [19]	Komoike, Y [20]	Runowicz, CD [21]	Paluch- Shimon, S [22]	Greenlee, H [23]	Senkus, E [24]
Domain												
Domain 1 Scope and Purpose	94.4%	100%	16.6%	100%	33.3%	55.5%	55.5%	72.2%	94.4%	88.8%	88.8%	83.3%
Domain 2 Stakeholder Involvement	61.%	66.6%	66.6%	27.7%	55.5%	88.8%	0%	50%	77.7%	50%	72.2%	27.7%
Domain 3 Rigour of Development	31.2%	58.3%	70.8%	29.16%	35.2%	52%	54.16%	33.3%	68.7%	33%	77.08%	35.4%
Domain 4 Clarity of Presentation	94.4%	100%	88.8%	94.4%	88.8%	27.7%	94.4%	100%	100%	100%	100%	100%
Domain 5 Applicability	70.8%	70.8%	66.6%	58.3%	25%	45.8%	20.8%	16.6%	62.5%	0%	50%	16.6%
Domain 6 Editorial Independence.	0%	0%	0%	0%	0%	50%	50%	50%	50%	50%	50%	0%
Total	58.6%	75.7%	51.5%	51.5%	39.6%	53.3%	30%	53.6%	75.5%	53.6%	73%	43.8%
Global Evaluation	5	6	5	5	4	5	4	4	6	5	6	4

%: Domain scores are calculated by adding all the points of the individual domain items and standardizing the total, as a percentage of the maximum possible score for that domain using the following formula: (Score obtained − Minimum possible score) / (Maximum possible score − Minimum possible score) × 100.

**Table 4:** Levels of Evidence and degrees of recommendation of the therapeutic physical exercise to reduce the adverse effects of breast cancer treatment, shown in the clinical practice guidelines (CPGs).

CPG	Doyle, C [13]	Rooney, M [14]	Manchon, P [15]	Murray, N [16]	Gradishar, WJ [17]	Harris, SR [18]	Greenlee, H [19]	Komoike, Y [20]	Runowicz, CD [21]	Paluch- Shimon, S [22]	Greenlee, H [23]	Senkus, E [24]
Treatment Aim												
Mortality reduction	D **											
Recurrence risk reduction	C *				**A ^†^**							
Increase quality of life	**B ^†^**	A *										
Pain reduction							B **		A **			
Fatigue reduction	A **	**B ^†^**				C **			A **		B **	
Lymphedema reduction	**B ^†^**					**D ^†^**			B **			
Musculoskeletal changes reduction									B **			
Increase in range of movement of the shoulder								A **				
Reduction of body weight alterations	**A ^†^**	**A ^†^**							**B ^†^**			B *
Reduction of bone alterations	**D ^†^**					**D**			**A ^†^**	**C ^†^**		
Reduction of gastrointestinal disorders	**		*									
Hot flashes reduction					**D ^†^**							
Neuropathy reduction									C *			
Stress							B *					
Depression		**B ^†^**					B *				B *	*
Anxiety							B *				B *	*

* Very recommendable, ** Moderately recommendable, **^†^** Controversy.

## References

[1] Colditz G.A., Bohlke K., Berkey C.S. (2015). Breast cancer risk accumulatio starts early-Prevention must also. Breast Cancer Res. Treat..

[2] Aebi S., Davidson T., Gruber G., Cardoso F. (2011). Primary breast cancer: Esmo clinical practice guidelines for diagnosis, treatment and follow-up. Ann. Oncol..

[3] Dos Santos W.D.N., Gentil P., de Moraes R.F., Ferreira Júnior J.B., Campos M.H., de Lira C.A.B., Freitas Júnior R., Bottaro M., Vieira C.A. (2017). Chronic Effects of Resistance Training in Breast Cancer Survivors. Biomed. Res. Int..

[4] Reis A.D., Pereira P.T.V.T., Diniz R.R., de Castro Filha J.G.L., Dos Santos A.M., Ramallo B.T., Filho F.A.A., Navarro F., Garcia J.B.S. (2018). Effect of exercise on pain and functional capacity in breast cancer patients. Health Qual. Life Outcomes.

[5] Williams A.D., Bird M.L., Hardcastle S.G., Kirschbaum M., Ogden K.J., Walters J.A. (2018). Exercise for reducing falls in people living with and beyond cancer. Cochrane Database Syst. Rev..

[6] Sweegers M.G., Altenburg T.M., Brug J., May A.M., van Vulpen J.K., Aaronson N.K., Arbane G., Bohus M., Courneya K.S., Daley A.J. (2018). Effects and moderators of exercise on muscle strength, muscle function and aerobic fitness in patients with cancer: A meta-analysis of individual patient data. Br. J Sports Med..

[7] Cantarero-Villanueva I., Fernández-Lao C., Cuesta-Vargas A.I., Del Moral-Avila R., Fernández-de-Las-Peñas C., Arroyo-Morales M. (2013). The effectiveness of a deep water aquatic exercise program in cancer-related fatigue in breast cancer survivors: A randomized controlled trial. Arch. Phys. Med. Rehabil..

[8] Galiano-Castillo N., Ariza-García A., Cantarero-Villanueva I., Fernández-Lao C., Díaz-Rodríguez L., Arroyo-Morales M. (2014). Depressed mood in breast cancer survivors: Associations with physical activity, cancer-related fatigue, quality of life, and fitness level. Eur. J. Oncol. Nurs..

[9] Fernández-Lao C., Cantarero-Villanueva I., Ariza-Garcia A., Courtney C., Fernández-de-las-Peñas C., Arroyo-Morales M. (2013). Water versus land-based multimodal exercise program effects on body composition in breast cancer survivors: A controlled clinical trial. Support Care Cancer.

[10] Trollope H., Leung J.P.Y., Wise M., Farquhar C., Sadler L. (2018). An evaluation of the objective quality and perceived usefulness of maternity clinical practice guidelines at a tertiary maternity unit. Aust. New Zeal. J. Obstet. Gynaecol..

[11] Brouwers M., Browman G.P., Burgers J. (2009). INSTRUMENTO AGREE II. Instrumento para la evaluación de Guías de práctica clínica. Heal San Fr. https://www.agreetrust.org/wp-content/uploads/2013/06/AGREE_II_Spanish.pdf.

[12] Harbour R., Miller J. (2001). A new system for grading recommendations in evidence based guidelines. BMJ.

[13] Doyle C., Kushi L.H., Byers T., Courneya K.S., Demark-Wahnefried W., Grant B., McTiernan A., Rock C.L., Thompson C., Gansler T. (2006). 2006 Nutrition, Physical Activity and Cancer Survivorship Advisory Committee; American Cancer Society (2006) Nutrition and physical activity during and after cancer treatment: An American Cancer Society guide for informed choices. CA Cancer J. Clin..

[14] Rooney M., Wald A. (2007). Interventions for the management of weight and body composition changes in women with breast cancer. Clin. J. Oncol. Nurs..

[15] Manchon P., Borràs J.M., Ferro T., Espinàs J.A. (2010). Breast Cancer OncoGuia Group (2010) Breast Cancer OncoGuia. Clin. Transl. Oncol..

[16] Murray N., Winstanley J., Bennett A., Francis K. (2009). Guideline Development Group. Diagnosis and treatment of advanced breast cancer: Summary of NICE guidance. BMJ.

[17] Gradishar W.J., Anderson B.O., Balassanian R., Blair S.L., Burstein H.J., Cyr A., Elias A.D., Farrar W.B., Forero A., Giordano S.H. (2018). Breast Cancer, Version 4.2017, NCCN Clinical Practice Guidelines in Oncology. J. Natl. Compr. Canc. Netw..

[18] Harris S.R., Schmitz K.H., Campbell K.L., McNeely M.L. (2012). Clinical practice guidelines for breast cancer rehabilitation: Syntheses of guideline recommendations and qualitative appraisals. Cancer.

[19] Greenlee H., Balneaves L.G., Carlson L.E., Cohen M., Deng G., Hershman D., Mumber M., Perlmutter J., Seely D., Sen A. (2014). Society for Integrative Oncology. Clinical practice guidelines on the use of integrative therapies as supportive care in patients treated for breast cancer. J. Natl. Cancer Inst..

[20] Komoike Y., Inokuchi M., Itoh T., Kitamura K., Kutomi G., Sakai T., Jinno H., Wada N., Ohsumi S., Mukai H. (2015). Japanese Breast Cancer Society. Japan Breast Cancer Society clinical practice guideline for surgical treatment of breast cancer. Breast Cancer.

[21] Runowicz C.D., Leach C.R., Henry N.L., Henry K.S., Mackey H.T., Cowens-Alvarado R.L., Cannady R.S., Pratt-Chapman M.L., Edge S.B., Jacobs L.A. (2016). American Cancer Society/American Society of Clinical Oncology Breast Cancer Survivorship Care Guideline. J. Clin. Oncol..

[22] Paluch-Shimon S., Pagani O., Partridge A.H., Bar-Meir E., Fallowfield L., Fenlon D., Friedman E., Gelmon K., Gentilini O., Geraghty J. (2016). Second international consensus guidelines for breast cancer in young women (BCY2). Breast.

[23] Greenlee H., DuPont-Reyes M.J., Balneaves L.G., Carlson L.E., Cohen M.R., Deng G., Johnson J.A., Mumber M., Seely D., Zick S.M. (2017). Clinical practice guidelines on the evidence-based use of integrative therapies during and after breast cancer treatment. CA Cancer J. Clin..

[24] Senkus E., Kyriakides S., Ohno S., Penault-Llorca F., Poortmans P., Rutgers E., Zackrisson S., Cardoso F. (2015). ESMO Guidelines Committee. Primary breast cancer: ESMO Clinical Practice Guidelines for diagnosis, treatment and follow-up. Ann. Oncol..

[25] Meneses-Echávez J.F., Correa-Bautista J.E., González-Jiménez E., Schmidt Río-Valle J., Elkins M.R., Lobelo F., Ramírez-Vélez R. (2016). The Effect of Exercise Training on Mediators of Inflammation in Breast Cancer Survivors: A Systematic Review with Meta-analysis. Cancer Epidemiol. Biomarkers Prev..

[26] Kyu H.H., Bachman V.F., Alexander L.T., Mumford J.E., Afshin A., Estep K., Veerman J.L., Delwiche K., Iannarone M.L., Moyer M.L. (2016). Physical activity and risk of breast cancer, colon cancer, diabetes, ischemic heart disease, and ischemic stroke events: Systematic review and dose-response meta-analysis for the Global Burden of Disease Study 2013. BMJ.

[27] Galloway D.A., Laimins L.A. (2016). Human papillomaviruses: Shared and distinct pathways for pathogenesis. HHS Public Access.

[28] Cheema B.S., Kilbreath S.L., Fahey P.P., Delaney G.P., Atlantis E. (2014). Safety and efficacy of progressive resistance training in breast cancer: A systematic review and meta-analysis. Breast Cancer Res. Treat..

[29] Tollosa D.N., Tavener M., Hure A., James E.L. (2019). Compliance with Multiple Health Behaviour Recommendations: A Cross-Sectional Comparison between Female Cancer Survivors and Those with no Cancer History. Int. J. Environ. Res. Public Health..

[30] Van Veen M.R., Mols F., Bours M.J.L., Weijenberg M.P., Kampman E., Beijer S. (2019). Adherence to the World Cancer Research Fund/American Institute for Cancer Research recommendations for cancer prevention is associated with better health-related quality of life among long-term colorectal cancer survivors: Results of the PROFILES registry. Support Care Cancer..

[31] Dennison R.A., Ward R.J., Griffin S.J., Usher-Smith J.A. (2019). Women’s views on lifestyle changes to reduce the risk of developing Type 2 diabetes after gestational diabetes: A systematic review, qualitative synthesis and recommendations for practice. Diabet Med..

[32] Adriouch S., Lelong H., Kesse-Guyot E., Baudry J., Lampuré A., Galan P., Hercberg S., Touvier M., Fezeu L.K. (2017). Compliance with Nutritional and Lifestyle Recommendations in 13,000 Patients with a Cardiometabolic Disease from the Nutrinet-Santé Study. Nutrients.

[33] Schmitz K.H., Campbell A.M., Stuiver M.M., Pinto B.M., Schwartz A.L., Morris G.S., Ligibel J.A., Cheville A., Galvão D.A., Alfano C.M. (2019). Exercise is medicine in oncology: Engaging clinicians to help patients move through cancer. CA Cancer J. Clin..

[34] Haskell W.L., Lee I.M., Pate R.R., Powell K.E., Blair S.N., Franklin B.A., Macera C.A., Heath G.W., Thompson P.D., Bauman A. (2007). Physical activity and public health: Updated recommendation for adults from the American College of Sports Medicine and the American Heart Association. Med. Sci. Sports Exerc..

[35] Igarashi Y., Akazawa N., Maeda S. (2018). Regular aerobic exercise and blood pressure in East Asians: A meta-analysis of randomized controlled trials. Clin. Exp. Hypertens..

[36] Espíndula R.C., Nadas G.B., Rosa M.I., da Foster C., Araújo F.C., de Grande A.J. (2017). Pilates for breast cancer: A systematic review and meta-analysis. Rev. Assoc. Med. Bras..

[37] Puşcaş D.M., Tache S. (2015). The importance of an exercise program in breast cancer related lymphedema. Palestrica of the Third Millennium- Civilization and Sport.

[38] Ramírez K., Acevedo F., Herrera M.E., Ibáñez C., Sánchez C. (2017). Actividad física y cáncer de mama: Un tratamiento dirigido. Rev. Med. Chil..

[39] Bruce J., Williamson E., Lait C., Richmond H., Betteley L., Lall R., Petrou S., Rees S., Withers E.J., Lamb S.E. (2018). Randomised controlled trial of exercise to prevent shoulder problems in women undergoing breast cancer treatment: Study protocol for the prevention of shoulder problems trial (UK PROSPER). B.M.J. Open.

[40] García-Hermoso A., Cavero-Redondo I., Ramírez-Vélez R., Ruiz J.R., Ortega F.B., Lee D.C., Martínez-Vizcaíno V. (2018). Muscular Strength as a Predictor of All-Cause Mortality in an Apparently Healthy Population: A Systematic Review and Meta-Analysis of Data From Approximately 2 Million Men and Women. Arch. Phys. Med. Rehabil..

[41] Mottola M.F., Davenport M.H., Ruchat S.M., Davies G.A., Poitras V.J., Gray C.E., Jaramillo Garcia A., Barrowman N., Adamo K.B., Duggan M. (2018). Canadian guideline for physical activity throughout pregnancy. Br. J. Sports Med..

